# NRXN1 as a novel potential target of antibody-drug conjugates for small cell lung cancer

**DOI:** 10.18632/oncotarget.27718

**Published:** 2020-09-29

**Authors:** Takuma Yotsumoto, Keita Maemura, Kousuke Watanabe, Yosuke Amano, Yoko Matsumoto, Koichi Zokumasu, Takahiro Ando, Masanori Kawakami, Hidenori Kage, Jun Nakajima, Yutaka Yatomi, Takahide Nagase, Daiya Takai

**Affiliations:** ^1^Department of Thoracic Surgery, The University of Tokyo Graduate School of Medicine, Tokyo, Japan; ^2^Department of Respiratory Medicine, The University of Tokyo Graduate School of Medicine, Tokyo, Japan; ^3^Department of Clinical Laboratory, The University of Tokyo Hospital, Tokyo, Japan

**Keywords:** antibody-drug conjugates, small cell lung cancer, novel molecular targets, NRXN1, cell adhesion molecule

## Abstract

Small cell lung cancer (SCLC) is a high-grade malignancy, and treatment strategies have not changed for decades. In this study, we searched for novel targets for antibody-drug conjugate (ADC) therapy for SCLC. We identified transmembrane proteins overexpressed specifically in SCLC with little or no expression in normal tissues and decided to focus on the cell adhesion molecule neurexin-1 (NRXN1). The cell surface overexpression of NRXN1 was confirmed using flow cytometry in SCLC cell lines (SHP77 and NCI-H526). The combination of a primary anti-NRXN1 monoclonal antibody and a secondary ADC exhibited anti-tumor activity in SCLC cell lines. Moreover, the knockout of NRXN1 in SHP77 cells resulted in a loss of the anti-tumor activity of NRXN1-mediated ADC therapy. Thus, NRXN1 could be a novel target for ADC therapy for the treatment of SCLC that is worth further research.

## INTRODUCTION

Small cell lung cancer (SCLC) accounts for 10–15% of lung cancer, and its prognosis has remained relatively dismal for years [1]. Most patients have metastatic spread at the time of diagnosis [2]. Currently, conventional platinum-based chemotherapy regimens with or without radiation remain the standard first-line treatment for SCLC. Although atezolizumab was approved for use in combination with carboplatin and etoposide as a first-line treatment for adult patients with extensive-stage SCLC, the median overall survival period, compared with that for chemotherapy alone, was only prolonged for a few months [3]. On the other hand, the role of surgery has been limited to rare (less than 5% of patients) for early-stage disease [4]. Although SCLC is more responsive to initial cytotoxic chemotherapy than non-small cell lung cancer, most patients relapse with a relatively resistant disease.

Genome-wide sequencing studies of SCLC have failed to identify targetable driver mutations such as EGFR, ALK, ROS1, and BRAF that are frequently observed in lung adenocarcinoma. Recurrent mutations of SCLC include the loss of the tumor suppressors TP53 and RB1, inactivating mutations in NOTCH family genes, and the amplification of MYC family genes, all of which are difficult to target [5]. The loss of PTEN, activating PI3K mutations, and aurora kinase activation have been reported as potential therapeutic targets [6]. There are ongoing trials for small molecule inhibitors of poly-ADP-ribose polymerase (PARP) [7–9] and an enhancer of zeste homolog 2 (EZH2), which regulate the DNA damage response and chromatin modifications, respectively [10]. A recent study proposed a new model of SCLC subtypes defined by the differential expressions of four key transcription regulators, ASCL1, NeuroD1, YAP1, and POU2F3, which would help to accelerate therapeutic research leading to targeted approaches [11]. Novel therapeutic modalities for SCLC are long awaited.

Antibody-drug conjugates (ADCs) are an emerging technology that has already been implemented in clinical practice for some malignancies. An ADC is a monoclonal antibody conjugated with a cytotoxic drug via a chemical linker, enabling selective drug delivery by binding to specific cell surface proteins [12, 13]. Considering the high sensitivity of SCLC to chemotherapy, the selective delivery of a cytotoxic agent using ADC could be a novel treatment strategy for SCLC [14].

Five ADCs have been approved by the Food and Drug Administration: brentuximab vedotin for Hodgkin lymphoma [15], ado-trastuzumab emtansine for HER2-positive metastatic breast cancer [16, 17], inotuzumab ozogamicin for acute lymphoblastic leukemia [18], gemtuzumab ozogamicin for CD33-positive acute myeloid leukemia [19], and trastuzumab deruxtecan for unresectable or metastatic HER2-positive breast cancer patients who have received two or more prior anti-HER2-based regimens in a metastatic setting [20]. To date, ADCs targeting solid tumors other than metastatic breast cancer have not exhibited distinct clinical benefits [21–29]. In SCLC, DLL3, a cell surface Notch ligand that appear to be a direct downstream target of ASCL1 [30, 31], has been identified as a novel target for ADCs [32]. However, a phase III trial comparing rovalpituzumab tesirine with topotecan as a second-line therapy had to be halted because of a shorter overall survival period in the ADC arm [33]. Trop-2, a glycoprotein overexpressed in many epithelial cancers, has also been reported to be a candidate target of ADCs [34, 35]. Sacituzumab govitecan, a Trop-2-targeting ADC, showed a potential efficacy and was deemed safe in a phase I/II trial in SCLC patients [36]. A phase I/II study of a CD56-targeting ADC in combination with carboplatin and etoposide showed no improvement in efficacy over standard carboplatin and etoposide therapy in SCLC [37]. Recently, promiximab-duocarmycin, a new CD56-targeting ADC, was shown to demonstrate promising activity in a preclinical study of SCLC.

In this study, we aimed to identify novel molecular targets for ADCs in SCLC. The candidates were cell surface proteins overexpressed specifically in tumors with little or no expression in normal tissues. We searched for transmembrane proteins of SCLC using a computational-biological approach. We herein report that NRXN1-mediated ADC exhibited anti-tumor activity *in vitro*, and thus NRXN1 could be a novel target of ADCs for SCLC.

## RESULTS

### mRNA expression profile of cell surface proteins in SCLC

We analyzed the expressions of cell surface proteins using microarray data available in the Cancer Cell Line Encyclopedia using an unsupervised clustering analysis. Among the 565 genes coding membrane proteins, 31 genes showed an increased compensated fluorescence signal by three times or more, on average. The National Center for Biotechnology Information (NCBI) RNA sequencing data was used to select genes with little or no expression in normal tissues. Interestingly, SCLC cell lines were divided into two subgroups with and without overexpression of the 31 genes. Since Rudin *et al*. reported a new model of SCLC subtypes based on the expressions of four key transcription regulators (ASCL1, NeuroD1, YAP1, and POU2F3) [11], we compared the results of our clustering analysis with their subtypes (Supplementary Figure 1). The NRXN1-positive SCLC cell lines generally overlapped with ASCL1-high or NEUROD1-high subtypes.

### NRXN1 expression in cell lines, surgical specimens, and human normal tissues

**Figure 1:**
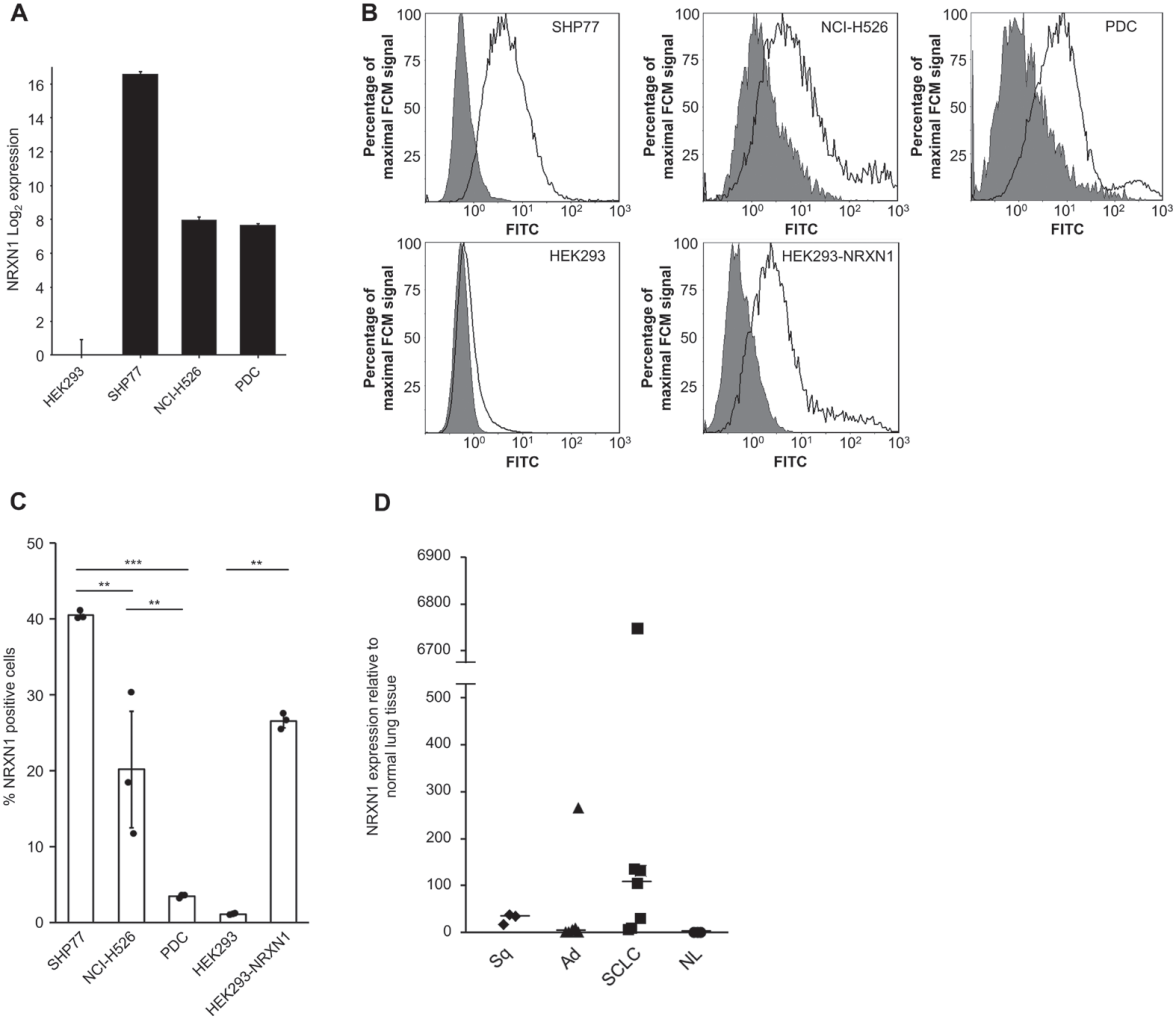
NRXN1 expression in cell lines and surgical specimens of lung tissue including non-SCLC and normal tissues. (**A**) The relative expressions in cell lines were examined using qRT-PCR with the SYBR green dye assay; data for NRXN1 is shown. The log_2_-scale relative gene expression is indicated on the *y*-axis. Error bars, SD. (**B**) Flow cytometry of NRXN1 on cell lines. Cell surface NRXN1 was assessed using FITC-conjugated anti-rabbit polyclonal antibody following rabbit anti-NRXN1 (Black trace) or IgG isotype control (gray-filled) antibodies. FCM, flow cytometry. (**C**) Percentage of NRXN1-positive cells examined for each cell line using flow cytometry. Cells were stained with rabbit anti-NRXN1 polyclonal antibody followed by FITC conjugated goat anti-rabbit IgG. A one-way analysis of variance (ANOVA) followed by the Tukey test was performed. (^*^ *P* < 0.05; ^**^ *P* < 0.01; ^***^ *P* < 0.0001; Tukey test). Error bars, SD. (**D**) Relative expression of NRXN1 in surgical specimens including normal lung by qRT-PCR using the SYBR green dye assay. The y-axis shows the NRXN1 expression levels relative to normal lung tissue. The horizontal bars indicate the median gene expression levels for each group. Error bars, SD. Ad, adenocarcinoma. NL, normal lung. SCLC, small cell lung cancer. Sq, squamous cell carcinoma.

Among the 31 membrane proteins, we focused on NRXN1. NRXN1 expression was analyzed using two SCLC cell lines (SHP77 and NCI-H526) and HEK293 cells. SHP77 had the highest mRNA expression, NCI-H526 displayed moderate expression, and HEK293 showed little expression (Figure 1A). Patient-derived cells (PDC) showed a moderate NRXN1 expression level that was slightly lower than that in the NCI-H526 cell line. Cell-surface NRXN1 protein expression was verified using flow cytometry (Figure 1B). The percentage of NRXN1-positive cells determined using flow cytometry is generally correlated with mRNA expression (Figure 1C). An analysis of the surgical specimens revealed a high NRXN1 expression in a subset of primary SCLCs (Figure 1D).

**Figure 2:**
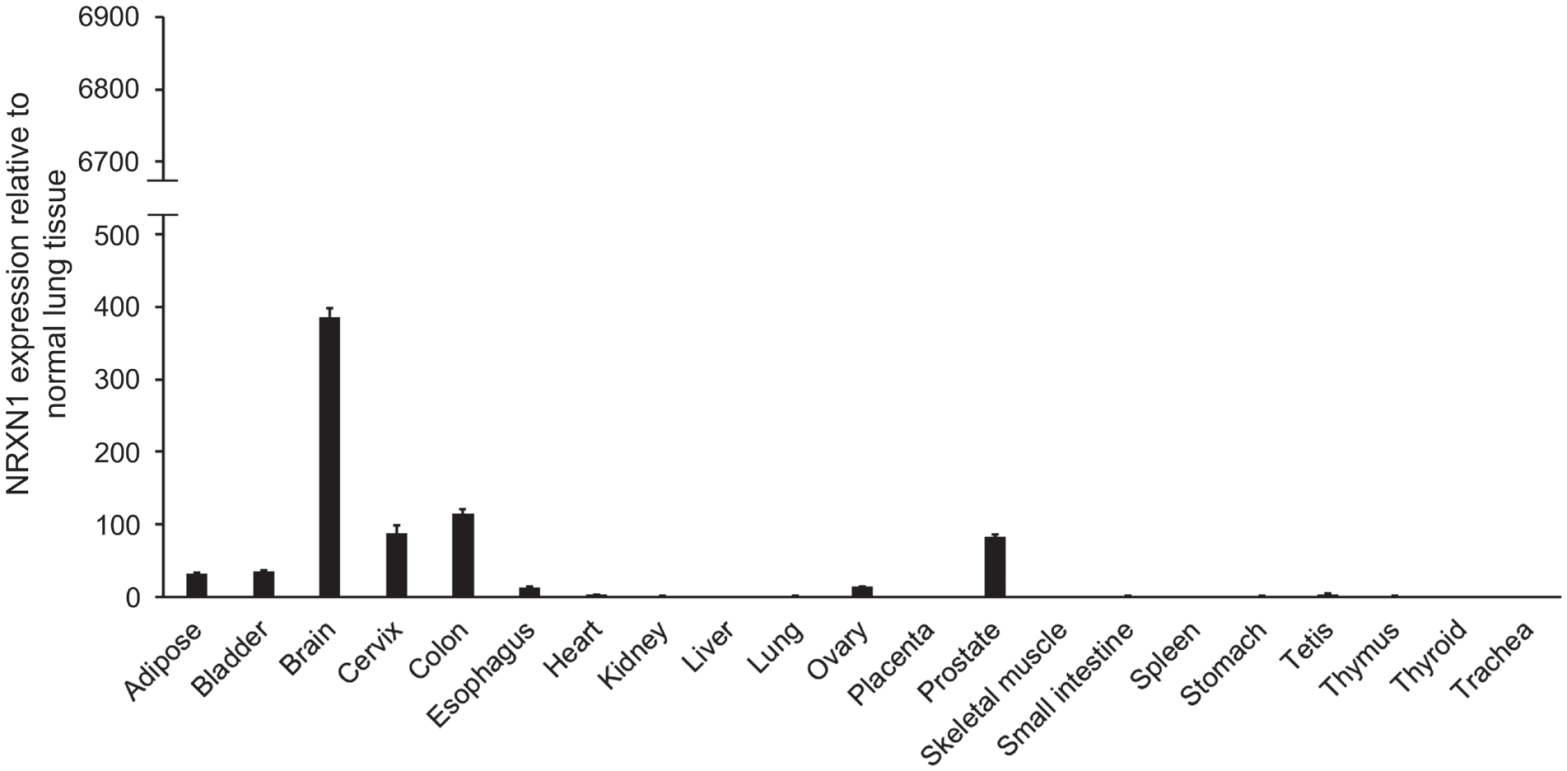
NRXN1 expression in normal multiple organs in humans. The relative expressions of NRXN1 in normal multiple organs of human tissue were examined using qRT-PCR with the SYBR green dye assay. The y-axis shows the NRXN1 expression levels relative to normal lung tissue. The y-axis is set to the same scale as that of Figure 1D so that the expression intensities of both surgical specimens and normal organs can be visually compared. Error bars, SD.

NRXN1 expression in a commercially available human RNA panel was also analyzed to confirm the minimal or absence of NRXN1 expression in normal tissues. Consistent with the NCBI RNA sequencing data, NRXN1 expression was relatively limited to the brain. In other normal tissues, the expression of NRXN1 was less than one-third of the level observed in the brain (Figure 2).

### Growth inhibition using a primary anti-NRXN1 monoclonal antibody and a secondary ADC

**Figure 3:**
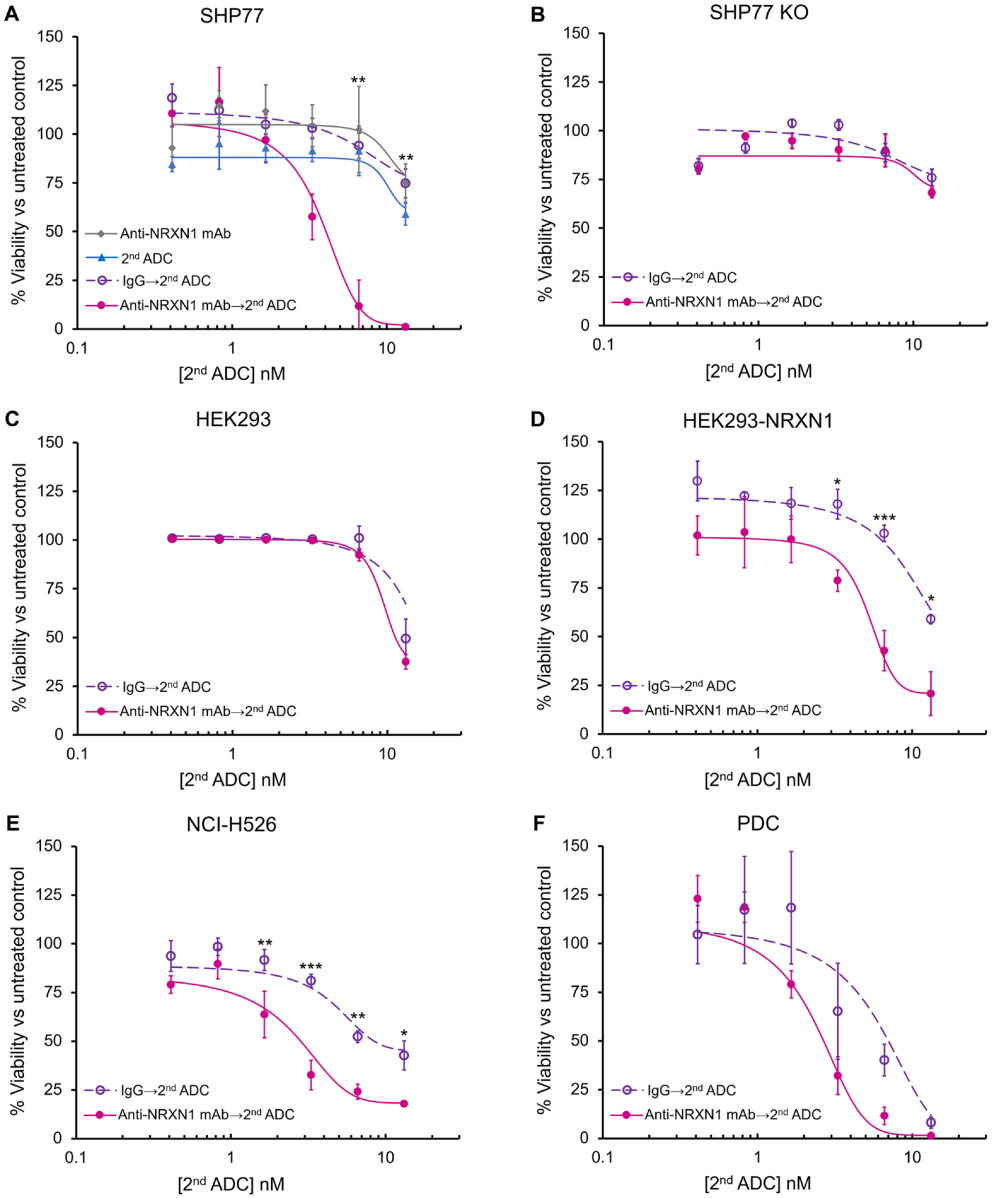
*In vitro* growth inhibition of NRXN1-targeted ADC. (**A**–**F**) *In vitro* growth inhibition of anti-NRXN1 monoclonal antibody only, secondary ADC only, isotype control antibody (IgG) followed by secondary ADC, and anti-NRXN1 monoclonal antibody followed by secondary ADC on incubation with (A) SHP77, (B) SHP77 KO, (C) HEK293, (D) HEK293-NRXN1, (E) NCI-H526, and (F) PDC. All the assays were performed in triplicate. A two-way ANOVA followed by the Tukey test was performed to assess the difference between IgG with the second ADC group and anti-NRXN1 mAb with the second ADC group. A *P* value < 0.05 was considered significant (^*^ *P* < 0.05; ^**^ *P* < 0.01; ^***^ *P* < 0.0001). Error bars represent the SD of the mean. mAb, Monoclonal antibody. ns, Not significant.

To screen the ADC activity, we used a monoclonal antibody against NRXN1 and a common secondary ADC capable of binding the primary antibody. In SHP77 cells, the secondary ADC caused a dose-dependent cell growth inhibition in the presence of anti-NRXN1 monoclonal antibody, whereas anti-NRXN1 monoclonal antibody alone, secondary ADC alone, or an IgG isotype control plus the secondary ADC did not inhibit cell growth (Figure 3A). The inhibitory concentration 50 (IC_50_) value of the secondary ADC in the presence of anti-NRXN1 monoclonal antibody was 3.8 nM, and the potency was more than 3-fold higher than that in the presence of the IgG isotype control antibody. On the other hand, NRXN1-defficient SHP77 cells showed little susceptibility to the secondary ADC with anti-NRXN1 monoclonal antibody (Figure 3B). NRXN1 overexpression sensitized HEK293 cells to the primary anti-NRXN1 monoclonal antibody and the secondary ADC (Figure 3C and 3D). In NCI-H526, growth inhibition by the secondary ADC in the presence of anti-NRXN1 monoclonal antibody was still observed, but to a lesser extent compared to that in SHP77 (Figure 3E). The PDC displayed reduced cell growth by secondary ADC, similar to that in the SHP77 cells, even in the presence of the IgG isotype control (Figure 3F).

Collectively, these results demonstrate that secondary ADC with anti-NRXN1 monoclonal antibody mediated cytotoxicity in a NRXN1 expression-dependent manner.

### Induction of apoptosis by the primary anti-NRXN1 monoclonal antibody and the secondary ADC

**Figure 4:**
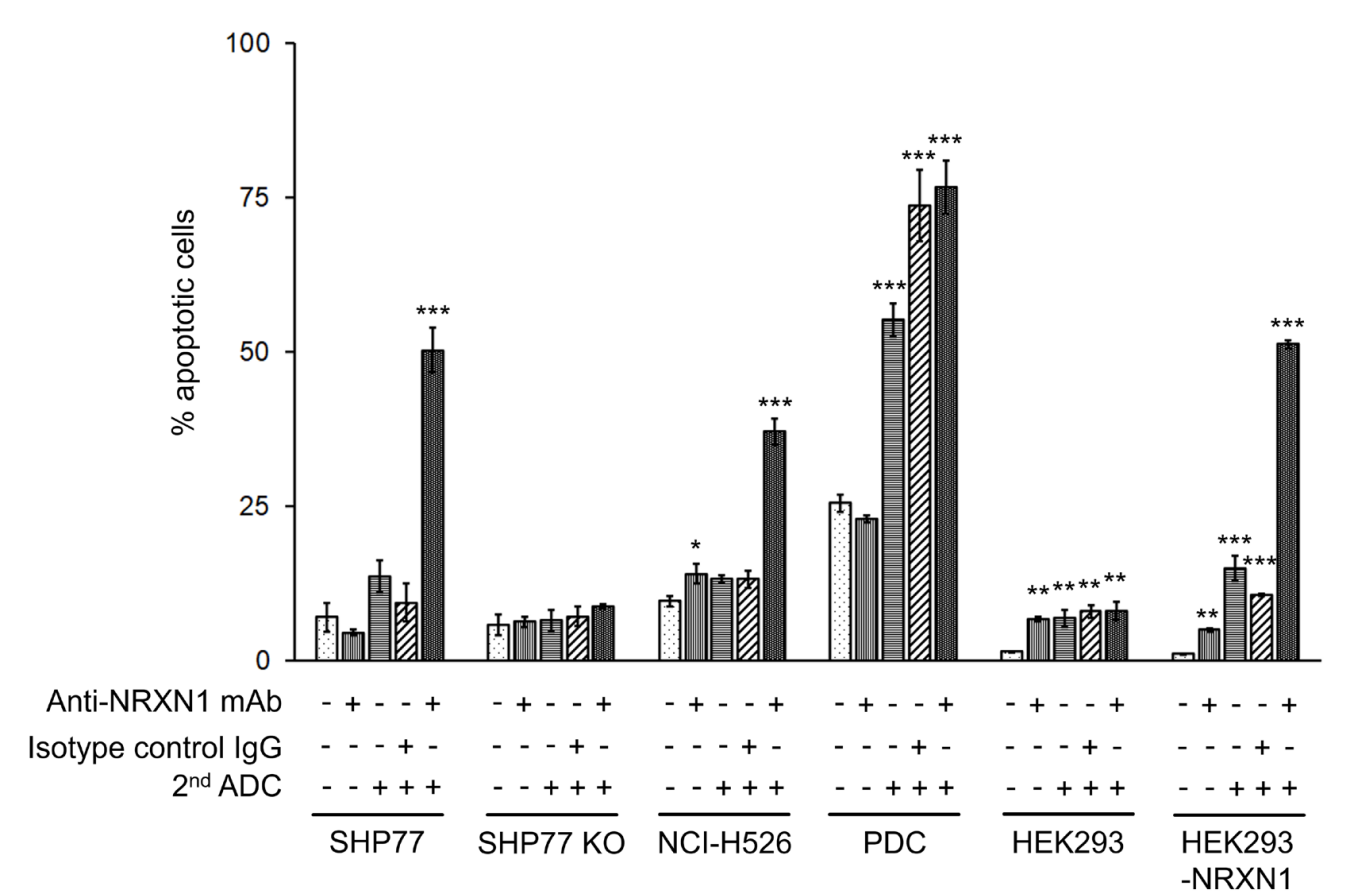
Apoptosis assay of NRXN1-targeted ADC at IC_50_ dose calculated by growth inhibition curves. Late apoptotic cells were quantified by Cy7-conjugated annexin-V and PI using flow cytometry. Results were analyzed using a one-way ANOVA followed by the Dunnett multiple comparisons test (^*^ *P* < 0.05; ^**^ *P* < 0.01; ^***^ *P* < 0.0001 versus no-treatment control group; Dunnett test). Error bars represent the SD of the mean. mAb, monoclonal antibody.

To verify actual cell death induced by secondary ADC with anti-NRXN1 monoclonal antibody, cells were treated with secondary ADC plus anti-NRXN1 monoclonal antibody, anti-NRXN1 monoclonal antibody alone, secondary ADC alone, and secondary ADC plus an IgG isotype control. Consistent with the growth inhibition assay shown in Figure 3A, apoptosis was significantly induced in SHP77 cells by the combination of the anti-NRXN1 monoclonal antibody and the secondary ADC (Figure 4). The induction of the apoptosis of PDC was also observed by secondary ADC alone or secondary ADC plus the IgG isotype control, suggesting that PDC had a high sensitivity to a low level of cytotoxic drug (PNU-159682) degraded from the secondary ADC.

## DISCUSSION

We identified 31 membrane proteins as candidates for novel ADC targets and demonstrated that NRXN1 is a promising target for ADCs in SCLC.

Neurexins are single-pass transmembrane proteins encoded by three genes (NRXN1, 2, and 3), and they function as cell adhesion molecules in synaptic transmission. Neurexins expressed in the presynaptic terminal bind postsynaptic neuroligins in a Ca^2+^-dependent manner, and the neurexin/neuroligin complex plays an important role in synaptic transmission [38]. The relationship between genomic alterations in NRXN genes and a wide variety of neuropsychiatric disorders, including autism spectrum disorders and schizophrenia, has been described in medical literature [39].

Each NRXN gene has at least two alternative promoters. An upstream promotor generates a longer α-isoform and a downstream promotor generates a shorter β-isoform. Moreover, multiple alternative splicing in both isoforms generates thousands of variants [40, 41], and it is difficult to strictly prove the selectivity of the used antibodies. As for the ADC experiments, the loss of cytotoxicity by specific NRXN1 knockout supported the selectivity of the primary antibody. In the flow cytometry, the loss of NRXN1 expression by specific NRXN1 knockout also provided evidence for the selectivity of the antibody.

To reduce the toxicity of ADC therapy, the expressions of the ADC target should be low or absent in normal tissues. NRXN1 is expressed in a limited manner in the central nervous system (CNS) and is a favorable target, since ADCs are unable to pass through the blood-brain barrier because of their large molecular size. A CD56-mediated ADC did not induce obvious damage to the CNS in xenograft models [42]. However, given that antineuronal autoantibodies are detected in paraneoplastic neurological syndrome, possible CNS side effects should be further validated using *in vivo* experiments.

Given the fact that NRXN1-mediated ADC exhibited favorable cytotoxic activity, monoclonal antibodies directly bound to cytotoxic agents should be generated and optimized for further study. Specifically, the use of different cytotoxic agents, cleavable or non-cleavable linkers, the optimal drug-antibody ratio, and epitopes should be explored and integrated comprehensively using many candidate antibodies. A smaller molecular size using a single antibody instead of two would contribute to more efficient drug delivery. The optimized ADCs should achieve their maximum effect while exhibiting a lower IC_50_ than that reported in this study through improvements in specific binding, efficient internalization, degradation and the potency of the cytotoxic agent.

Recently, a new nomenclature for SCLC subtypes based on the expressions of four key transcription regulators was reported [11]. NRXN1-positive cell lines generally overlapped with ASCL1-high or NeuroD1-high subtypes, suggesting that NRXN1-positive cell lines belong to subtypes with enhanced neuroendocrine characteristics (Supplementary Figure 1). This result is convincing, considering that NRXN1 is expressed at synapses in the CNS. As to NRXN1 expression in SCLC, not all cases of SCLC are NRXN1-positive, and the proportion of NRXN1-positive cases remains unclear. We tested commercially available anti-NRXN1 polyclonal antibodies (ANR-031 from Alomone Labs and ab214191 from Abcam) for immunohistochemistry using SCLC tissue microarrays, but they lacked specificity (data not shown). Still, the results of our study suggest that an NRXN1-enriched SCLC group could benefit from NRXN1-mediated ADCs.

In the study, we did not directly verify the internalization and trafficking of anti-NRXN1 monoclonal antibody with secondary ADC. We could not conjugate sensor or fluorescent dyes directly to the secondary ADC, because the chemical information of the secondary ADC was a proprietary information of the manufacturer. Moreover, using the third antibody conjugated to sensor or fluorescent dyes to detect the secondary ADC would alter its behavior due to the enlarged size of the complex. In addition, the host species of the secondary ADC was also undisclosed by the manufacturer. Although the results of cell growth inhibition and the apoptosis assay in the present study imply the internalization of the anti-NRXN1 antibody with the secondary ADC because of the significant anti-tumor effects that were observed, the internalization, efficiency of the internalization, and intracellular trafficking of NRXN1-mediated ADCs should be addressed in future research using optimized monoclonal antibodies directly conjugated to payloads.

In conclusion, we identified NRXN1 as a new target for ADCs by screening membrane proteins using a computational-biological approach. The combination of the primary anti-NRXN1 monoclonal antibody and the secondary ADC exhibited anti-tumor activity in an NRXN1-expression dependent manner. NRXN1 could be a novel potential target of ADCs for SCLC that is worth further research.

## MATERIALS AND METHODS

### *In silico* selection of new potential targets for ADCs

We exported the gene expression profiles of 51 SCLC cell lines available at the Cancer Cell Line Encyclopedia and those of 30 normal lung tissue samples examined using the Human Genome U133 Plus 2.0 Array (Thermo Fisher Scientific, Waltham, MA) at the Gene Expression Omnibus (GEO) of the NCBI (Supplementary Table 1). Genes coding membrane proteins were identified using Gene Ontology (GO) and its subontology, known as the Cellular Component Ontology, with the GO terms “plasma membrane” (GO: 0005886) and “anchored component of membrane” (GO: 0031225) or “integral component of membrane” (GO: 0016021).

### Cell lines and clinical samples

SCLC cell lines were obtained from the American Type Culture Collection and were cultured according to the manufacturer’s instructions. SHP77 and H526 cells were cultured in Roswell Park Memorial Institute (RPMI 1640) medium with L-glutamine and phenol red (Wako Pure Chemical Industries, Osaka, Japan) containing 10% fetal bovine serum (FBS, Biowest, Nuaillé, France) and 5% Antibiotic-Antimycotic Mixed Stock Solution (Nacalai Tesque, Kyoto, Japan). HEK293 and HEK293-NRXN1 cells were cultured in Dulbecco’s modified Eagle’s medium (DMEM) with L-glutamine and phenol red (Wako Pure Chemical Industries) containing 10% FBS and 5% Antibiotic-Antimycotic Mixed Stock Solution (Nacalai Tesque). All the cells were cultured at 37°C in a humidified incubator with 5% CO_2_ and passaged every 3 to 4 days. As to patient-derived cells, the circulating tumor cells of an 84-year-old male with SCLC were derived from his peripheral blood after the approval of the Institutional Review Board at the University of Tokyo Hospital. The circulating tumor cells were purified using RosetteSep™ CTC Enrichment Cocktail Containing Anti-CD56 (STEMCELL Technologies, Vancouver, Canada) according to the manufacturer’s protocol. The purified cells were cultured in RPMI-1640 supplemented with bFGF (20 μg/L, #13256029, Thermo Fisher Scientific), EGF (20 μg/L, #PHG0314, Thermo Fisher Scientific), and B-27™ supplement (#17504044, Thermo Fisher Scientific) using Coster 24 Well Clear Flat Bottom Ultra Low Attachment Multiple Well Plates (Corning, Corning, NY) [43].

### Generation of NRXN1 KO cells

We knocked out the NRXN1 gene using the lentiviral CRISPR/Cas9 sgRNA mediated expression knockout protocol. Candidates for the sgRNA were configured using the CRISPR design tool CHOPCHOP [44] and CRISPRdirect [45]. Selected candidates for the sgRNA (Merck, Darmstadt, Germany) were cloned into Cas9 SmartNuclease All-in-one Vector (System Biosciences, Palo Alto, CA). The insert coding Cas9 and the sgRNA was ligated into the lentivirus vector CSII-CMV-MCS-IRES2-Bsd (RIKEN BioResource Research Center, Tsukuba, Japan) after being removed from the Cas9 SmartNuclease All-in-one Vector. The SHP77 cells underwent lentiviral transduction using polybrene. After incubation for 14 days with medium containing 10 μg/mL blasticidin, the cell populations were screened using flow cytometry and qRT-PCR to confirm the efficiency of the CRISPR knockout of NRXN1 (Supplementary Figure 2).

### mRNA expression analysis

RNA was extracted using RNAiso plus (TAKARA BIO, Shiga, Japan). Complementary DNA (cDNA) was generated from 1 μg of RNA using SuperScript III Reverse Transcriptase (Thermo Fisher Scientific). Each cDNA and primers specific for NRXN1 (forward primer 5′- GAT TCT TAC CAC AAC GGG CTA CA-3′, and reverse primer 5′-GGG TTT CAA AGG TGA TTG GGT C-3′) and GAPDH (forward primer 5′-CAC CAC CAA CTG CTT AGC AC-3′, and reverse primer 5′-TGG CAG GTT TTT CTA GAC GG-3′) were mixed with THUNDERBIRD SYBR qPCR Mix (TOYOBO, Osaka, Japan). The reaction mixes were run on the 7500 Fast Real-Time PCR System (Thermo Fisher Scientific). Relative gene expression was calculated using the ddCt method. NRXN1 expression in human normal tissues was examined using he FirstChoice Human Total RNA Survey Panel (Thermo Fisher Scientific).

### Antibodies

Mouse anti-NRXN1α monoclonal antibody (sc-136001, Santa Cruz Biotechnology, Dallas, TX) recognizing amino acids 1063-1184 of rabbit NRXN1α was applied to the cell viability assay. Anti-NRXN1α polyclonal antibody raised in rabbits (ANR-031; Alomone Labs, Jerusalem, Israel) against amino acid residues 546-560 of rat NRXN1α was applied for NRXN1 measurements using flow cytometry. The secondary ADC used in the study was αMFc-CL-PNU (AM-102-PN; Moradec, San Diego, CA), an anti-mouse IgG Fc-specific antibody conjugated to PNU-159682 with a cleavable linker. PNU-159682 is a derivative of nemorubicin, which induces cell death by intercalating DNA and topoisomerase inhibition. The antibody portion is a polyclonal antibody specific to the Fc region of mouse IgGs. The cleavable linker is stable in extracellular fluid, but is cleaved by cathepsin in endosomes once the conjugate is internalized into cells.

### NRXN1 overexpression

cDNA was generated from RNA extracted from SHP77 cells. The DNA sequence coding NRXN1 was reconstructed by polymerase reaction with the first half and the latter half of the entire coding sequence [46]. The first half of the sequence was amplified with forward primer 5′-TCC CGC CTT TCC CCT TAC-3′ and reverse primer 5′-GCT GGA ATT ACA GTT AAT CCT GAT AC-3′. The latter half of the sequence was amplified with forward primer 5′-GGA GCA TGT TTA TGA AAA TTC AG-3′ and reverse primer 5′-CAT TCC CTG TCT TCT TTT GTA TG-3′. The full-length sequence was subcloned to the pGEM-T Easy Vector plasmid (Promega, Madison, WI). The plasmid was transformed into *E. coli* DH-5α Competent Cells (TaKaRa Bio) according to the manufacture’s protocol. The sequences of plasmids from colonies of the competent cells were verified after being extracted from transformed DH-5α using the PureYield™ Plasmid Miniprep System (Promega, Madison, WI). The collected plasmids were extracted from competent cells incubated overnight using the PureYield^™^ Plasmid Midiprep System (Promega). Fragments including the NRXN1 sequence were subcloned into pcDNA™3.1/Zeo^(+)^(Thermo Fisher Scientific). HEK293 cells were transfected with HilyMax Reagent (Dojindo, Kumamoto, Japan) and incubated for 2 days, at which time the cells were examined to confirm the overexpression of the target genes using qRT-PCR and flow cytometry.

### Flow cytometry

Cells were incubated with 2.5 μL of rabbit anti-NRXN1 polyclonal antibody (ANR-031; Alomone Labs) or an isotype control at 1 × 10^5^ cells/100 μL in PBS with 2% FBS for 30 min in the dark. After two washes, the cells were incubated with 2 μL of goat anti-rabbit IgG FITC conjugate (ab97050; Abcam, Cambridge, United Kingdom) for 30 min.

After two washes, 2 μL of PI (Biolegend, San Diego, CA) were added to the cells for 15 min before the FC500 flow cytometer (Beckman Coulter, Brea, CA) run. Data were analyzed using FC500 analysis software (Beckman Coulter).

### *In vitro* cytotoxic assay

On day 1, the cancer cells, HEK293, and HEK293-NRXN1 cells were seeded at a density of 1000 cells per well in 50 μL of medium on 96-well culture plates. PDCs were plated at a density of 2500 cells per well. On day 2, the primary antibodies (anti-NRXN1 monoclonal antibody or IgG isotype control; 50 μL per well) were added to each well. After incubation in the presence of the primary antibodies for 10 min, 2 μL of the secondary ADC was added as a serial dilution. The primary antibody (anti-NRXN1 monoclonal antibody or IgG isotype control) and the secondary ADC were added at a constant volume ratio of 2:1 in each well. The cell lines and PDCs were incubated for 3 and 7 days, respectively, and were subjected to a cell growth assay using the Cell Counting Kit-8 (Dojindo).

### Apoptosis assay

Apoptosis was analyzed using double staining with Annexin-V Cy7 conjugate (BioLegend) and PI (BioLegend) according to the manufacturer’s instructions. Briefly, cells were incubated in Annexin-V binding buffer. After incubation with 5 μL of Annexin-V Cy7 conjugate and 2 μL of PI for 15 min, the cells were examined using FC500. Data were analyzed using FC500 software.

### Statistical methods

Data are presented as the mean ± SD, as stated in the figure legends. The statistical analysis of the gene expression profiles obtained from CCLE and NCBI GEO was performed using R (version 3.1.1, R Foundation for Statistical Computing) and Bioconductor. The statistical analysis of the NRXN1-positive cells detected using flow cytometry, the *in vitro* growth inhibition assay, and the apoptosis assay was performed using JMP Pro 14.2 software (SAS Institute Inc., Cary, NC). The results of the NRXN1-positive cells detected using flow cytometry were analyzed using a one-way ANOVA followed by the Tukey test (^*^ *P* < 0.05; ^**^ *P* < 0.01; ^***^ *P* < 0.0001; Tukey test). Regarding the *in vitro* growth inhibition assay, a two-way ANOVA followed by the Tukey test was performed for multiple comparison analyses of the *in vitro* cytotoxic activity (^*^ *P* < 0.05; ^**^ *P* < 0.01; ^***^ *P* < 0.0001; Tukey test). The percentage of late apoptotic cells (Annexin V and PI double-positive cells) were compared with those of untreated cells using a one-way ANOVA followed by the Dunnett test (^*^ *P* < 0.05; ^**^ *P* < 0.01; ^***^ *P* < 0.0001 versus no-treatment control group; Dunnett test). Differences were considered significant when the *P* value was less than 0.05.

## SUPPLEMENTARY MATERIALS



## Abbreviations

ADC: antibody-drug conjugate
ALK: anaplastic lymphoma kinase
ANOVA: analysis of variance
ASCL1: achaete-scute homolog 1
bFGF: basic fibroblast growth factor
Cas9: CRISPR-associated proteins 9
cDNA: complementary deoxyribonucleic acid
CRISPR: clustered regularly interspaced short palindromic repeats
CSC: cancer stem cell
Cy: cyanine
DLL3: delta-like ligand 3
EGF: epidermal growth factor
EGFR: epidermal growth factor receptor
EZH2: enhancer of zeste homolog 2
FITC: fluorescein isothiocyanate
GABA: gamma-aminobutyric acid
GAPDH: Glyceraldehyde 3-phosphate dehydrogenase
HER2: erb-b2 receptor tyrosine kinase 2
KRAS: kirsten rat sarcoma viral oncogene homologue
mAb: monoclonal antibody
MYC: cellular myelocytomatosis oncogene
NEUROD1: Neurogenic differentiation 1
NRXN: neurexin
PARP: poly-ADP- ribose polymerase
PBS: phosphate buffered saline
PI: propidium iodide
PI3K: phosphoinositide 3 kinase
POU2F3: POU class 2 homeobox 3
PTEN: phosphatase and tensin homologue deleted on chromosome 10
RB1: retinoblastoma
SCLC: small cell lung cancer
TP53: tumor suppressor protein p53
TROP2: Trophoblast cell surface antigen 2
YAP1: Yes-associated protein 1
