# Supplementary material for: NRXN1 as a novel potential target of antibody-drug conjugates for small cell lung cancer
Source: Oncotarget. 2020 Sep 29;11(39):3590–600. doi: 10.18632/oncotarget.27718 (PMC7533074; doi:10.18632/oncotarget.27718)
Supplement: Supplementary file 1 [file oncotarget-11-3590-s001.pdf]

NRXN1 as a novel potential target of antibody-drug conjugates for small cell lung cancer

SUPPLEMENTARY MATERIALS

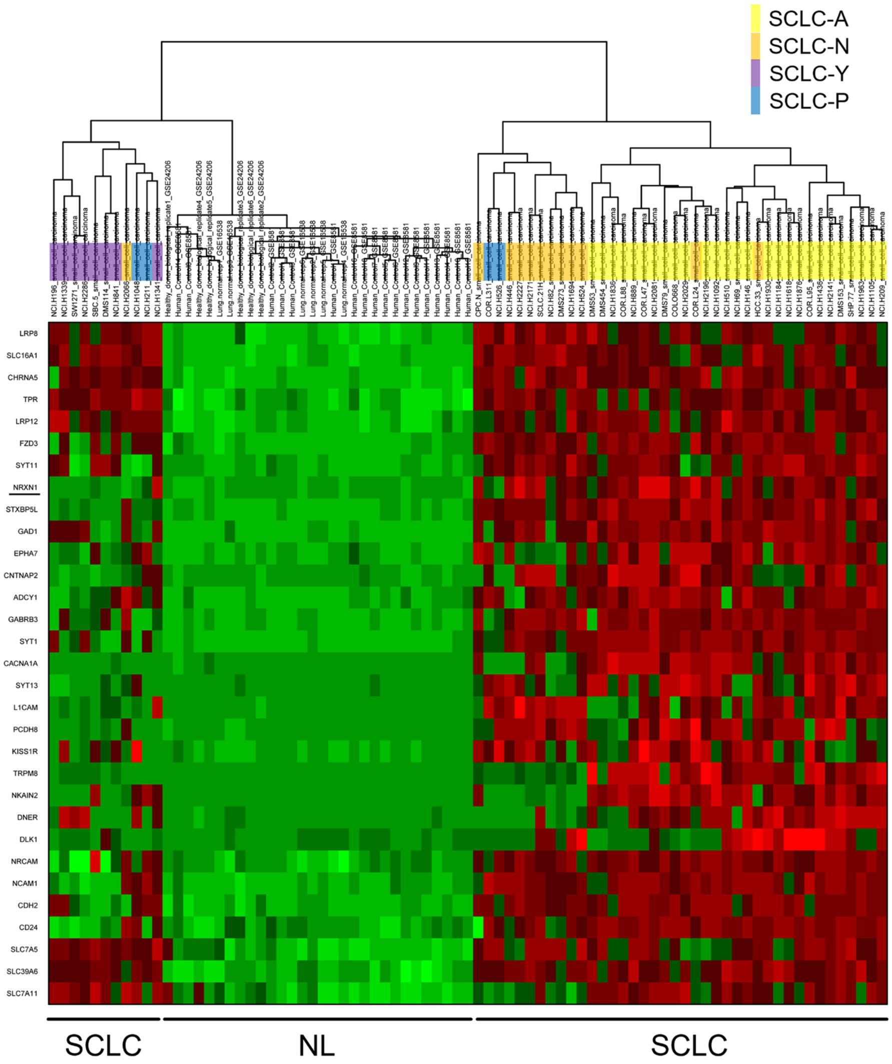

Supplementary Figure 1: *In silico* supervised cluster analysis of genes with higher expressions in SCLC cell lines compared to human normal lung tissue samples. The signal intensity acquired from the microarray analysis is represented using a color scale (red, higher expression; green, lower expression). NRXN1 is underlined on the vertical axis. Note that the SCLC cell lines were divided into two groups at an early phase of the clustering analysis. The color bars indicate the four SCLC subtypes of the SCLC cell lines as reported by Rudin *et al.* [11]. Color codes are indicated at the upper right of the figure. The last letter of SCLC-A, SCLC-N, SCLC-Y, SCLC-P signifies the transcription regulator most strongly associated with each subtype. A, ASCL1. N, NeuroD1. Y, YAP1. P, POU2F3. SHP77 and NCI-H526 adopted in the study are classified as SCLC-A type and SCLC-P type, respectively.

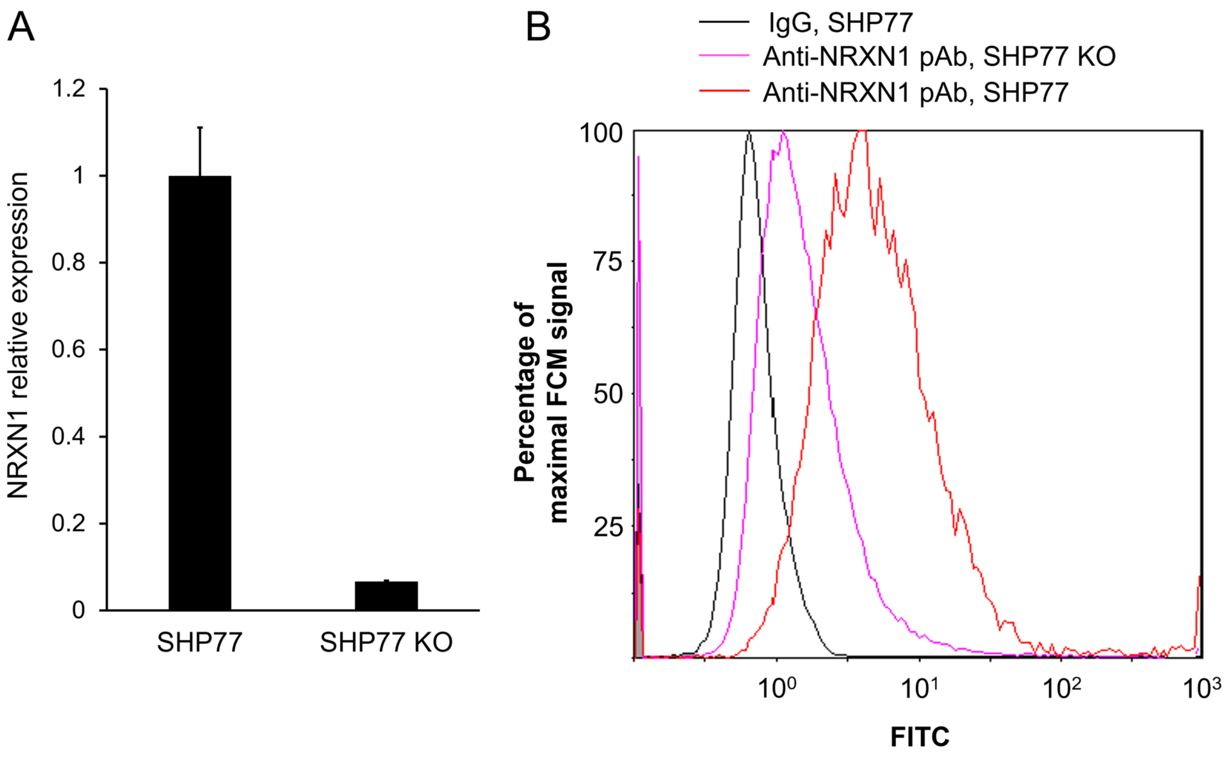

Supplementary Figure 2: Characterization of SHP77 and SHP77 KO cells. (**A**) Relative NRXN1 expression of SHP77 and SHP77 KO cells was examined using qRT-PCR with the SYBR green dye assay. Error bars, SD. (**B**) Flow cytometry of NRXN1 on SHP77 and SHP77 KO cells. Cell surface NRXN1 was assessed using rabbit anti-NRXN1 polyclonal antibody followed by FITC-conjugated anti-rabbit polyclonal antibody. FCM, flow cytometry. pAb, polyclonal antibody.

Supplementary Table 1: List of GEO data sets and samples for analysis of gene expression

GEO dataset#	GEO sample#	Description	
GSE8581	GSM210005	Human_Control1	
GSE8581	GSM210009	Human_Control2	
GSE8581	GSM210014	Human_Control3	
GSE8581	GSM210015	Human_Control4	
GSE8581	GSM210192	Human_Control5	
GSE8581	GSM210196	Human_Control18	
GSE8581	GSM210979	Human_Control6	
GSE8581	GSM211008	Human_Control7	
GSE8581	GSM212067	Human_Control8	
GSE8581	GSM212068	Human_Control9	
GSE8581	GSM212070	Human_Control10	
GSE8581	GSM212787	Human_Control11	
GSE8581	GSM212789	Human_Control12	
GSE8581	GSM212790	Human_Control13	
GSE8581	GSM212811	Human_Control14	
GSE8581	GSM212853	Human_Control15	
GSE8581	GSM213035	Human_Control16	
GSE8581	GSM213036	Human_Control17	
GSE16538	GSM415386	Lung-normal-rep1	
GSE16538	GSM415387	Lung-normal-rep2	
GSE16538	GSM415388	Lung-normal-rep3	
GSE16538	GSM415389	Lung-normal-rep4	
GSE16538	GSM415390	Lung-normal-rep5	
GSE16538	GSM415391	Lung-normal-rep6	
GSE24206	GSM595407	Healthy donor_biological replicate1	
GSE24206	GSM595411	Healthy donor_biological replicate2	
GSE24206	GSM595414	Healthy donor_biological replicate3	
GSE24206	GSM595416	Healthy donor_biological replicate4	
GSE24206	GSM595417	Healthy donor_biological replicate5	
GSE24206	GSM595419	Healthy donor_biological replicate6	
GSE36139	GSE36133	COLO668	SCLC cell line
GSE36140	GSE36134	COR-L24	SCLC cell line
GSE36141	GSE36135	COR-L311	SCLC cell line
GSE36142	GSE36136	COR-L47	SCLC cell line
GSE36143	GSE36137	COR-L88	SCLC cell line
GSE36144	GSE36138	COR-L95	SCLC cell line
GSE36145	GSE36139	CPC-N	SCLC cell line
GSE36146	GSE36140	DMS114	SCLC cell line
GSE36147	GSE36141	DMS153	SCLC cell line
GSE36148	GSE36142	DMS273	SCLC cell line
GSE36149	GSE36143	DMS454	SCLC cell line
GSE36150	GSE36144	DMS53	SCLC cell line
GSE36151	GSE36145	DMS79	SCLC cell line
GSE36152	GSE36146	HCC-33	SCLC cell line
GSE36153	GSE36147	NCI-H1048	SCLC cell line
GSE36154	GSE36148	NCI-H1092	SCLC cell line
GSE36155	GSE36149	NCI-H1105	SCLC cell line

GSE36156	GSE36150	NCI-H1184	SCLC cell line
GSE36157	GSE36151	NCI-H1339	SCLC cell line
GSE36158	GSE36152	NCI-H1341	SCLC cell line
GSE36159	GSE36153	NCI-H1436	SCLC cell line
GSE36160	GSE36154	NCI-H146	SCLC cell line
GSE36161	GSE36155	NCI-H1618	SCLC cell line
GSE36162	GSE36156	NCI-H1694	SCLC cell line
GSE36163	GSE36157	NCI-H1836	SCLC cell line
GSE36164	GSE36158	NCI-H1876	SCLC cell line
GSE36165	GSE36159	NCI-H1930	SCLC cell line
GSE36166	GSE36160	NCI-H1963	SCLC cell line
GSE36167	GSE36161	NCI-H196	SCLC cell line
GSE36168	GSE36162	NCI-H2029	SCLC cell line
GSE36169	GSE36163	NCI-H2066	SCLC cell line
GSE36170	GSE36164	NCI-H2081	SCLC cell line
GSE36171	GSE36165	NCI-H209	SCLC cell line
GSE36172	GSE36166	NCI-H211	SCLC cell line
GSE36173	GSE36167	NCI-H2141	SCLC cell line
GSE36174	GSE36168	NCI-H2171	SCLC cell line
GSE36175	GSE36169	NCI-H2196	SCLC cell line
GSE36176	GSE36170	NCI-H2227	SCLC cell line
GSE36177	GSE36171	NCI-H2286	SCLC cell line
GSE36178	GSE36172	NCI-H446	SCLC cell line
GSE36179	GSE36173	NCI-H510	SCLC cell line
GSE36180	GSE36174	NCI-H524	SCLC cell line
GSE36181	GSE36175	NCI-H526	SCLC cell line
GSE36182	GSE36176	NCI-H69	SCLC cell line
GSE36183	GSE36177	NCI-H82	SCLC cell line
GSE36184	GSE36178	NCI-H841	SCLC cell line
GSE36185	GSE36179	NCI-H889	SCLC cell line
GSE36186	GSE36180	SBC-5	SCLC cell line
GSE36187	GSE36181	SCLC-21H	SCLC cell line
GSE36188	GSE36182	SHP-77	SCLC cell line
GSE36189	GSE36183	SW1271	SCLC cell line
